# Exploring Anti-Aging Effects of Topical Treatments for Actinic Keratosis

**DOI:** 10.3390/medicina61020207

**Published:** 2025-01-24

**Authors:** Federica Li Pomi, Andrea d’Aloja, Dario Valguarnera, Mario Vaccaro, Francesco Borgia

**Affiliations:** 1 Department of Precision Medicine in Medical, Surgical and Critical Care (Me.Pre.C.C.), University of Palermo, 90127 Palermo, Italy; federicalipomi@hotmail.it; 2 Department of Clinical and Experimental Medicine, Section of Dermatology, University of Messina, 98125 Messina, Italy; andredalo@hotmail.it (A.d.); dario96.v@gmail.com (D.V.); mario.vaccaro@unime.it (M.V.)

**Keywords:** actinic keratosis, anti-aging, oxidative stress, photodynamic therapy, skin aging, skin lightening, solar lentigo, tirbanibulin, 5-fluorouracil

## Abstract

*Background and Objectives*: Actinic keratosis (AK) is a precancerous cutaneous lesion driven by chronic ultraviolet (UV) exposure, often coexisting with features of photoaging, such as wrinkles and pigmentary irregularities. Recent evidence suggests that treatments for AK may also counteract photoaging through shared molecular pathways, including oxidative stress and inflammation. This narrative review explores the dual benefits of AK therapies, highlighting their potential anti-aging and skin-lightening effects, and implications for improving skin appearance alongside lesion clearance. *Materials and Methods*: The literature was analyzed to assess the efficacy, mechanisms, and cosmetic outcomes of commonly used AK treatments, including topical agents (5-fluorouracil (5-FU), imiquimod, diclofenac, and tirbanibulin), and photodynamic therapy (PDT). Studies highlighting their effects on photoaged skin, collagen remodeling, pigmentation, and patient satisfaction were reviewed. *Results*: PDT emerged as the most validated treatment, demonstrating improved collagen synthesis, skin texture, and pigmentation. 5-FU showed remodeling of the dermal matrix and increased procollagen levels, but local skin reactions represent a major limitation. Imiquimod enhanced dermal fibroplasia and reduced solar elastosis, while diclofenac provided mild photodamage improvements with minimal adverse effects. Tirbanibulin showed promising aesthetic outcomes, including skin lightening and a reduction in mottled pigmentation, with favorable tolerability. *Conclusions*: AK therapies offer a dual-purpose strategy, addressing both precancerous lesions and cosmetic concerns associated with photoaging. While PDT remains the gold standard, emerging agents like tirbanibulin ointment exhibit substantial potential. Future research should focus on optimizing treatment protocols and evaluating long-term cosmetic outcomes to enhance patient satisfaction and compliance.

## 1. Introduction

Actinic keratosis (AK) is a precancerous cutaneous lesion commonly arising on sun-exposed areas, such as the face, scalp, and extremities, particularly in fair-skinned individuals over the age of 40 [1]. AK pathogenesis follows a multistep process triggered by genetic mutations and inflammation induced by chronic ultraviolet (UV) radiation exposure. These UV-induced mutations induce epidermal homeostasis dysregulation, leading to abnormal keratinocyte proliferation and, in some cases, progression to cutaneous squamous cell carcinoma (cSCC) [2]. As hallmark features of photodamaged skin, AKs frequently coexist with other lesions linked to cumulative UV damage, encompassing both benign and malignant conditions, such as melanoma, basal cell carcinoma, and cSCC. Alongside these lesions, signs of prolonged UV exposure include wrinkles, irregular hyperpigmentation, such as solar lentigo (SL), and xerosis [3].

Currently, several topical treatments are approved for AK management, including 4% and 5% 5-fluorouracil (5-FU) cream, 0.5% 5-FU combined with 10% salicylic acid (5-FU/SA), 3% diclofenac sodium in hyaluronic acid (HA) gel, and 3.75% and 5% imiquimod (IMI) cream, tirbanibulin 1% ointment and photodynamic therapy (PDT) [4]. They aim not only to treat visible AK lesions but also to address the broader cancerization field, namely the area of subclinical UV-induced damage, that does not yet present visible clinical lesions. Such treatments, with different application schedules and mechanisms of action, are primarily aimed at normalizing keratinocyte proliferation and promoting epidermal turnover, thereby eliminating precancerous lesions. However, emerging evidence suggests that these therapies, in addition to their antineoplastic effects, may also offer cosmetic benefits by reducing the photoaging features commonly observed in AK patients.

Moving from these premises, we aimed to review the literature on the topic, exploring the dual therapeutic potential of AK treatments, focusing on their anti-aging and skin-lightening effects and their broader implications in enhancing skin appearance.

## 2. Materials and Methods

To narratively review the literature focused on the anti-aging effects of AK topical treatments, we evaluated English-language papers, using the databases PubMed, Scopus, and Web of Science.

The following Medical Subject Heading (MeSH) terms were used: “photodynamic therapy”, “5-fluorouracil”, “imiquimod”, “tirbanibulin”, “diclofenac”, which were individually associated with: “skin aging”, “anti-aging”, “skin-lightening”, “solar lentigo”, and “pigmentation”. The inclusion criteria targeted studies that evaluated the efficacy, mechanisms of action, and cosmetic outcomes of AK treatments, with a specific focus on their effects on photoaged skin, collagen remodeling, pigmentation, and patient satisfaction. Eligible articles provided either quantitative or qualitative data on the dual therapeutic potential of AK topical agents. The exclusion criteria ruled out studies focusing solely on oncological outcomes or those lacking relevant cosmetic or anti-aging data. No ethical approval was required for this review.

## 3. Results

### 3.1. 5-Fluorouracil Cream

5-FU is a pyrimidine analog that irreversibly inhibits thymidylate synthase, thus blocking DNA synthesis and inducing apoptosis in highly proliferative AK cells [5]. Currently, several 5-FU formulations are available, including 5% 5-FU cream, 4% fluorouracil cream, 5-FU/SA solution, and 5% fluorouracil plus calcipotriol 0.005% cream. The 5% 5-FU cream, applied twice daily for four consecutive weeks as a field treatment for facial AKs, achieved a 38% complete clearance rate (CCR) at 6 months in a large vehicle-controlled randomized trial, thus reducing the need for localized spot therapies for more than two years [6].

In confirming this, recent network meta-analyses, which compared the efficacy of 5% FU with other topical therapies, highlighted that 5% 5-FU performs better in reducing the total number of lesions [7,8]. Furthermore, 4% 5-FU in aqueous cream showed comparable efficacy to 5% 5-FU but with improved tolerability [9]. However, its main limitation remains the frequent occurrence of moderate to severe local skin reactions (LSRs) [9]. In contrast, in a randomized controlled trial, 5-FU/SA cream achieved a CCR of 49.5% for slightly palpable and moderately thick hyperkeratotic AKs at 8 weeks post-treatment, following a 12-week regimen. However, the occurrence of LSRs, closely linked to both treatment duration and efficacy, posed a significant challenge to patient compliance [10]. Regarding its anti-aging effects, 5% 5-FU therapy seems to cause epidermal injury, which triggers wound healing and subsequent remodeling of the dermal matrix, leading to an overall improvement in skin appearance. Sachs et al. investigated the effects of 5% 5-FU cream applied twice daily for 2 weeks on photodamaged facial skin in 21 AK patients, highlighting significant increases in markers of epidermal damage, inflammation, and extracellular matrix degradation (including matrix metalloproteinases (MMP) 1 and 3), 24 h post-treatment. By 4 weeks, mRNA levels of type I and III procollagen increased 7-fold and 3-fold, respectively, with type I procollagen protein levels doubling by week 24. Clinically, both AKs and photoaging signs improved significantly, with most participants reporting enhanced skin appearance and expressing the willingness to repeat the treatment [11]. The effectiveness of 5% 5-FU has also been demonstrated in treating photodamage of the upper limbs. A study involving 32 patients, comparing the efficacy of 5-FU in cream versus peel form, highlighted the anti-aging benefits of both formulations. Treatment achieved noticeable improvements in skin appearance and a reduction in dermal elastotic material. Biopsies revealed decreased levels of epidermal p53 and an increase in procollagen I levels, further supporting the rejuvenating effects of 5% 5-FU therapy [12]. On the other hand, no significant photoaging improvement was evidenced after using topical 5% 5-FU for non-melanoma skin cancer prevention in a secondary analysis of data from a randomized trial involving 932 veterans [13]. These conflicting data prevent us from reaching definitive conclusions about the effectiveness of 5-FU as an anti-aging therapeutical option.

**Figure 1:**
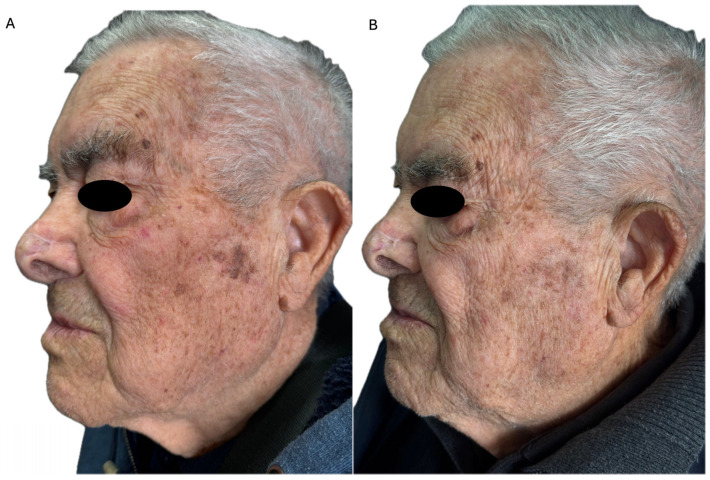
(**A**) A 92-year-old man with multiple facial actinic keratoses in the context of photodamaged skin, with wrinkles, mottled pigmentation and seborrheic keratoses before 5-fluorouracil 4% cream treatment; (**B**) almost complete clearance of the treated actinic keratoses, with amelioration of photodamage and reduction in seborrheic keratoses, at the 6-month follow-up.

Conversely, as far as we know, no studies have investigated the skin rejuvenation effects of 4% 5-FU cream, while only limited data are available about the anti-aging effects of 5-FU/SA. A recent Italian study highlighted the cosmetic improvement of a 77-year-old woman following treatment with 5-FU/SA cream, including complete resolution of AKs and significant amelioration in skin appearance [14]. To date, only one case report has highlighted the full resolution of seborrheic keratosis (SK) in a 65-year-old patient, following 4 weeks of therapy, characterized by the onset of severe LSR (Table 1) (Figure 1) [15]. 

### 3.2. Diclofenac 3% in Hyaluronic Acid

Diclofenac 3% in hyaluronic acid (HA) is a nonsteroidal anti-inflammatory drug (NSAID) with greater selectivity for the inducible cyclooxygenase-2 (COX-2) enzyme over the constitutive COX-1. By inhibiting COX-2, it reduces the production of pro-inflammatory prostaglandins, thereby decreasing inflammation and modulating the skin’s immune response. This may help normalize keratinocyte activity, reduce abnormal proliferation, and promote the regression of AK lesions. A key advantage of this formulation is its favorable safety profile, as it is generally easily tolerated with minimal LSRs. However, its efficacy can be limited, particularly in thick lesions. The combined results of two vehicle-controlled trials that included twice-daily ninety-day treatments indicated a CCR of 42% for patients receiving diclofenac, as opposed to 14% for those in the vehicle group, with CCR at a year varying between 18% and 29% [16,17].

Regarding anti-aging effects, a study on 20 patients with AKs and photodamaged skin evaluated its efficacy after 2 months of treatment, using clinical assessments and reflectance confocal microscopy (RCM). After treatment, significant clinical improvements in irregular pigmentation and skin roughness were observed, along with improvements in RCM features, suggesting that diclofenac in HA may improve both clinical and microscopic characteristics of photodamaged skin [18]. Regarding skin-lightening, Afify et al. reported a reduction in the area affected by SKs following therapy with diclofenac 1% in 30 patients, with a complete resolution of only one lesion [19]. Another anecdotal report described the complete clearance of SK in a 73-year-old man treated with diclofenac 3% applied twice daily for one month. However, the authors did not hypothesize the mechanism by which diclofenac acts on seborrheic lesions (Table 2) [20].

### 3.3. Imiquimod Cream

IMI cream is a topical immunomodulator that stimulates Toll-like receptor 7 (TLR7), triggering immune responses that promote the production of several cytokines, including interferon-alpha (IFN-α) and tumor necrosis factor-alpha (TNF-α). This enhanced immune response promotes the elimination of abnormal or injured cells, including AK precancerous cells [21]. The topical application of IMI cream in different formulations has demonstrated moderate-to-excellent efficacy in managing AKs across different anatomical sites [5]. IMI is available as a 5% cream to be applied three times weekly for 12–16 weeks or a 3.75% cream to be applied three times a week for 4 weeks. In vehicle-controlled studies, IMI 3.75% cream achieved CCRs ranging from 34% to 59.5% for the treatment of facial and scalp AKs [22]. Conversely, in a double-blind study evaluating the efficacy of 5% IMI, patients in the IMI group achieved an overall CCR of 55.0% compared to 2.3% in the vehicle group [23].

Currently, limited evidence supports the rejuvenating effects of IMI cream, especially in reducing solar elastosis and in the return of normal epidermal thickness [24]. A study evaluating pre- and post-therapy biopsies from 12 AK patients revealed significant histologic and immunohistologic changes in UV-damaged skin. Post-treatment observations revealed a reduction in hyperkeratosis, a more uniform rete ridge architecture, and a decreased presence of sun-damaged melanocytes in the epidermis. Additionally, there was an increase in cellularity within regions of solar elastosis [25]. These morphological changes suggest that IMI therapy promotes epidermal reorganization and dermal remodeling, finally resulting in skin appearance improvements. A prospective non-comparative pilot study highlighted that 72.7% of patients who applied IMI 5% cream to the periorbital skin reported a skin-rejuvenating effect, as assessed using a self-reported 5-point Likert scale [26]. Focusing on hyperpigmented lesions, the clearance of SLs in patients treated with imiquimod 3.75% for AKs and field cancerization has been anecdotally reported, with two case reports describing the lightening of SL following IMI 3.75% cream application [27,28]. Conversely, in a study comparing cryotherapy, calcipotriene, tazarotene, and imiquimod 5% cream in the treatment of SKs, the latter did not reach significant results (Table 3) [29].

### 3.4. Photodynamic Therapy

PDT is a non-invasive therapeutic procedure based on the combined use of a photosensitive drug, 5-aminolevulinic acid (ALA) or its methylated ester (MAL), and a light source, natural or artificial, of adequate wavelength, which causes necrosis and/or selective apoptosis of tumoral, inflammatory and infected cells, accounting for its broad spectrum of clinical applications [30,31,32]. Pooled data from three studies evaluating up to two sessions of red-light ALA-PDT demonstrated lesion clearance rates of 89.1% for AK patients treated with ALA-PDT and 32.7% for those receiving placebo-PDT at 12 weeks post-treatment [33,34,35]. However, a recent randomized trial comparing four treatment approaches—5% 5-FU, 5% imiquimod, ingenol mebutate, and MAL-PDT—revealed that MAL-PDT had a lower cumulative probability of remaining free from treatment failure compared to 5-FU, making it one of the least effective therapeutic options [7].

The rejuvenating effects of PDT are already reported in the latest European guidelines with recommendation strength A and quality of evidence I [32]. This places PDT a step ahead of other AK therapies due to its broad range of therapeutic applications [36]. PDT significantly improves many photoaged skin characteristics, including fine lines, mottled pigmentation, sallowness, skin texture, tactile roughness, and telangiectasia. However, the quality of evidence is generally low to moderate, with great variability between studies, especially for ALA-PDT [37]. To support its anti-aging efficacy, there are currently in vitro and in vivo studies on animals and humans, as well as on organ transplant recipients (OTR). 

In vitro, PDT has been demonstrated to stimulate type I collagen and the collagen-degrading enzyme MMP-3 and decrease elastotic material in the dermis, thus counteracting the signs of photoaging [36,38,39,40]. It has been proposed that elevated levels of MMP-3 may aid in the degradation and clearance of old, damaged collagen fibers, while fibroblasts contribute to the synthesis of new collagen to replace them. The interplay between epithelial and mesenchymal cells seems to play a pivotal role in PDT-induced rejuvenation, with keratinocytes promoting the release of cytokines that stimulate collagen production in dermal fibroblasts [41,42,43]. ALA-PDT also appears to affect the immune microenvironment of photo-aged skin. PDT restored antigen presentation and dendritic cell migration, enhancing immune cell–cell communication, suggesting that PDT may renew immune cells, reduce immunosenescence, and remodel the immune microenvironment in photo-aged skin, with implications on chronological and systemic aging as well [44]. 

Animal studies confirmed what emerged from in vitro ones. In mice, PDT was demonstrated to induce collagen renewal, improve skin inflammation and reduce signs of photoaging [45]. Low-dose ALA-PDT appears to act on lymphatic vessel remodeling in intrinsically aged skin of SKH-1 mice by improving lymphatic and blood vessel density and drainage function, thereby improving fluid balance and removing noxious substances [46]. As evidenced in vitro, low-dose PDT indirectly stimulates dermal collagen regeneration through keratinocyte–fibroblast interaction: it triggers keratinocytes to release TGF-β1, activating the TGF-β pathway in fibroblasts, thus finally promoting collagen synthesis and fibroblast proliferation [47]. 

Several case series and clinical trials on the skin rejuvenating effect of PDT are also reported, with great heterogeneity between photosensitizers, concentrations, incubation times, light sources, number of sessions and intervals between sessions. Furthermore, several studies have compared the efficacy of ALA–pulsed dye-laser (PDL) or ALA–intense pulsed light (IPL) with PDL or IPL alone, demonstrating in all cases that photosensitizer application improves performance for both the anti-neoplastic and the anti-aging effect (Table 4).

While considerable evidence supports the anti-aging effects of PDT, the same cannot be said for its skin-lightening effects. Kim et al. suggested PDT’s inhibitory effect on melanogenesis in human melanocytes. Interestingly, when exposed to melanocytes, keratinocytes or dermal fibroblasts inhibited melanogenesis through a paracrine effect, likely by reducing the release of melanocyte-stimulating cytokines. In vivo, PDT has been shown to reduce mottled hyperpigmentation in photoaged skin, highlighting its clinical potential for skin whitening [48]. In contrast, few studies highlighted PDT-induced pigmentation, with post-inflammatory hyperpigmentation being one of the most frequent side effects in clinical practice [49]. As regards the treatment of SKs, isolated case reports have shown a lightening effect on the lesions, often using combined therapies with surgical lasers [50,51,52].

**Table 4:** Studies on human beings in which PDT has demonstrated anti-aging effects. ALA: aminolevulinic acid; GAIS: Global Aesthetic Improvement Scale; IPL: intense pulsed light; LPNY: Nd:YAG laser. MAL: methyl aminolevulinic acid; OTR: organ transplant recipient; PDL: pulsed-dye laser; PDT: photodynamic therapy.

Authors	Treatment	Intervals	N° of Patients	Outcome
Zhang et al. [53]	10% ALA	Every 2 weeks	10	Improvement in fine line and mottled pigmentation assessed with global scores of photoaging
Huang et al. [54]	2% 5-ALA gel with HA and light-emitting diode—red light	3 treatments every 4 weeks	6	Skin improvement assessed with GAIS
Kohl et al. [55]	MAL-IPL vs. placebo-IPL	3 treatments at 6 weeks intervals	37	Improved photoaged skin
Sanclemente [56]	MAL-PDT (daylight) vs. placebo–PDT (daylight)	Sun exposure for 120 min in 3 sessions	48	Significant improvement after MAL-PDT
Zang et al. [57]	ALA 5% (2 h incubation) Red light vs. red-light PDT vs. IPL vs. IPL-PDT	1 session	10	Red-light PDT and IPL-PDT groups showed better results than IPL group
Shin et al. [58]	0.5% ALA liposomal spray with IPL-PDT vs. LPNY	3 treatments every 3 weeks	13	PDT-treated periorbital wrinkles showed better amelioration
Ji et al. [59]	ALA-PDT vs. red light alone	2 sessions	14	ALA-PDT group performed better than red-light group
Hasson et al. [60]	MAL-PDT	1 or 2 sessions	16 OTR	62.5% improvement in photodamage as measured by SkinCare^®^
Piccioni et al. [61]	0.5% liposome-encapsulated 5-ALA spray and IPL	3 sessions every 3 weeks	30	Periorbital and nasolabial wrinkle reduction
Palm et al. [62]	MAL: split face with blue and red light	1 session	18	No difference between the two light sources
Kosaka et al. [63]	ALA 5% IPL vs. IPL alone	3 sessions every 4 weeks	16	ALA-IPL performed better than IPL alone
Xi et al. [64]	ALA 5% IPL vs. IPL alone	3 sessions every 4 weeks	24	ALA-IPL performed better than IPL alone
Touma et al. [65]	ALA 20%; 0.5–1 h incubation; blue light	1 session	17	Significant improvement in fine lines, texture and dyspigmentation
Avram et al. [66]	ALA 20%; 1 h incubation; IPL	1 session	17	Amelioration in telangiectasias, mottled pigmentation and in coarseness of skin texture
Alster et al. [67]	ALA 20%; 1 h; split face ALA-IPL vs. IPL alone	2 sessions	10	ALA-IPL performed better than IPL alone
Dover et al. [68]	ALA 20%; 30–60 min; ALA-IPL vs. IPL alone	3 sessions every 3 weeks	20	ALA-PDL performed better than IPL alone
Gold et al. [69]	ALA 20%; 30–60 min incubation; ALA-IPL vs. IPL alone	3 sessions	13	ALA-IPL performed better than IPL alone
Zane et al. [70]	MAL-PDT; 3 h incubation	2 sessions	20	Improvement in pigmentation, fine wrinkles, coarseness
Bjerring et al. [71]	0.5% liposome-encapsulated ALA spray vs. 20% ALA-PDT	3 sessions every 3 weeks	37	Amelioration of periorbital and perioral wrinkles
Rui-Rodriguez et al. [72]	MAL-PDT; split face: 1 h incubation on one half and 3 h on the other half	3 sessions every 2 month	10	Moderate improvement in fine wrinkles and coarseness, mostly on the 3 h time side
Haddad et al. [73]	ALA 20%; 2 h; split face ALA-IPL vs. IPL alone	1 session	24	ALA-IPL performed better

### 3.5. Tirbanibulin Ointment

Tirbanibulin 1% ointment represents the latest arrival among the therapeutic options on the market, being approved for the treatment of non-hyperkeratotic AKs of the face and scalp. It is a selective inhibitor of tubulin polymerization, a protein essential for the functioning of the cytoskeleton and for cell division. It blocks the cell cycle of abnormal AK keratinocyte cells, thus promoting their apoptosis. In pivotal Phase 3 trials, tirbanibulin demonstrated good efficacy, achieving a CCR in 44–54% of patients, and excellent tolerability [74]. These findings are also supported by real-world data, showing comparable results [75,76,77,78]. A recent meta-analysis revealed that tirbanibulin demonstrates a favorable safety profile and efficacy comparable to the already available AK treatments, with its key advantage being the shorter treatment duration of just five consecutive days [79]. Recent real-world studies have expanded the potential applications of tirbanibulin beyond its established use for AKs. Emerging evidence suggests its efficacy in managing both tumoral and viral conditions such as basal cell carcinoma, Bowen’s disease, genital warts, and vulvar intraepithelial neoplasia, highlighting its versatility in dermatological and oncological fields [80,81,82].

**Figure 2:**
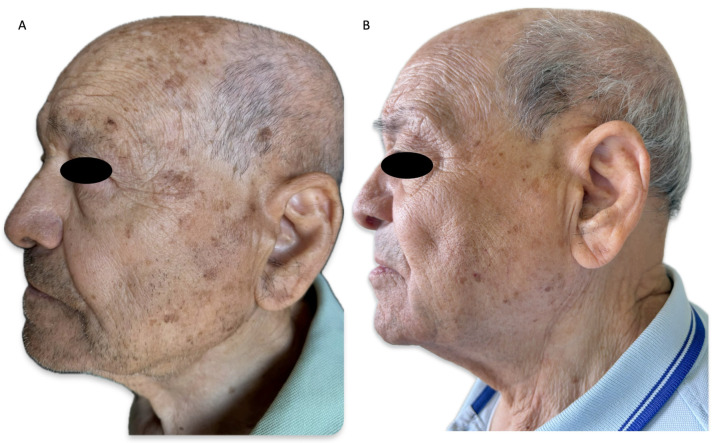
(**A**) An 82-year-old man with multiple facial actinic keratoses in the context of photodamaged skin, with wrinkles and mottled pigmentation before tirbanibulin 1% ointment treatment; (**B**) complete clearance of the treated actinic keratoses, with an amelioration of photodamage and reduction in solar lentigines, at 6-month follow-up.

Focusing on anti-aging effects, tirbanibulin is proving to be promising for possible use in the aesthetic field. A recent case report documented the skin-lightening effect of tirbanibulin ointment in a 78-year-old male with AKs and SL within the field of cancerization [83]. At 2-month follow-up, clearance of AKs and SL was detected in the treated area. Subsequently, a case series of seven patients highlighted the anti-aging effect of tirbanibulin together with a reduction in mottled pigmentation and SLs [84]. The same authors conducted a single-center study on a large case series of 42 patients affected by both AKs and SLs, highlighting a CCR in 35% of SLs and a PCR in 50% at 2-month follow-up. The stunning aspect of the study was the excellent maintenance of the aesthetic results at 6-month follow-up [85]. Finally, a single-arm study on 26 patients highlighted an improvement in skin quality measurements from baseline to 57 days, identified using UV fluorescence imaging (Table 5) (Figure 2) [22].

## 4. Discussion

Chronic exposure to UV radiation is the main trigger of both AKs and photoaging, which share a common pathogenesis rooted in cumulative photodamage. In AKs, UV-induced mutations in epidermal keratinocytes, particularly in genes involved in cellular homeostasis and DNA repair, including p53, lead to their dysregulated proliferation, which finally leads to the development of AK precancerous lesions. Specifically, UVA (315–400 nm) penetrates the dermis and induces damage indirectly through ROS production, leading to oxidative stress and inflammation. This inflammation involves macrophages, neutrophils, prostaglandins, and interleukins, creating a feedback loop that amplifies ROS and tissue damage. Conversely, UVB (280–315 nm) penetrates the basal epidermis and directly damages keratinocyte DNA by causing mutations in the TP53 gene, impairing the p53 protein’s role in DNA repair and apoptosis. UVB also promotes ROS generation, further exacerbating oxidative stress-related damage [3,86,87].

Chronic UV exposure accelerates skin aging, or “photoaging”, by increasing nicotinamide adenine dinucleotide phosphate hydrogen (NADPH) oxidase levels, which generate ROS. This overwhelms the cutaneous antioxidant defenses, resulting in the degradation of collagen and elastin, impaired collagen synthesis, and a disrupted dermal structure. Additionally, UVB-induced activation of MMPs further exacerbates collagen degradation, intensifying the damage. These cumulative effects result in the hallmark features of photoaging, such as reduced skin elasticity, skin atrophy, solar elastosis, impaired wound healing, and pigmentation disorders, including SLs [88,89].

The shared molecular and clinical features of AKs and skin aging highlight the interconnected nature of oxidative stress-induced skin damage, offering a strong rationale for investigating AK treatments for their potential anti-aging benefits. Given these overlapping pathways, therapies aimed at treating AKs may also prove effective in addressing photoaging. Such approaches could simultaneously target precancerous AK lesions while rejuvenating the skin by counteracting the structural and functional decline associated with aging, positioning AK treatments as a dual-purpose strategy for improving both skin health and appearance. 

From the literature review, it appears that the therapies currently available on the market demonstrate potential anti-aging effects; however, their efficacy, mechanisms, and practical applicability vary greatly. 

PDT stands out as the most extensively validated treatment for its dual role in addressing AK and reversing signs of photoaging [33]. Supported by evidence, PDT promotes dermal collagen remodeling through increased synthesis of type I collagen and degradation of elastotic material, as well as improvements in skin texture, pigmentation, and fine lines. The mechanism involves keratinocyte–fibroblast interaction and the stimulation of TGF-β1 release, which orchestrates collagen synthesis [37,39,40,41]. Its ability to treat broader areas with mild adverse effects and its inclusion in guidelines with high recommendation strength further cement its position as a preferred treatment for both AKs and cosmetic concerns. However, variability between studies emerges, with different photosensitizers used, different wavelengths, intervals between sessions, and the number of sessions.

5-FU offers promising, though less consistent, anti-aging benefits. Studies report significant remodeling of the dermal matrix, including a sevenfold increase in type I procollagen mRNA and doubled protein levels within weeks [12,13]. Clinical observations align with these findings, with notable improvements in photodamage and wrinkles. However, conflicting results from large-scale trials and the frequent onset of LSRs limit its broader application for cosmetic purposes. Lower concentrations of 4% 5-FU or combination therapies, such as 5-FU/SA, show potential for milder reactions, but data on their rejuvenating effects remain limited.

Imiquimod demonstrates moderate anti-aging effects through its immunomodulatory mechanism, which induces dermal fibroplasia and epidermal reorganization [25]. Histological evidence reveals reduced solar elastosis, restoration of normal epidermal thickness, and increased collagen synthesis [26]. Nevertheless, the associated inflammatory response and inconsistent efficacy in improving pigmentation limit its utility as a cosmetic therapy. Anecdotal reports of SL clearance warrant further investigation to confirm these findings in larger studies.

Diclofenac 3% in HA gel shows milder but measurable improvements in photodamage, including better skin texture and mottled pigmentation reduction [21]. Despite its favorable safety profile and tolerability, its lower efficacy in treating hyperkeratotic lesions and limited impact on collagen remodeling makes it less suitable for standalone anti-aging applications. However, its minimal side effects position it as a useful adjunct for patients unable to tolerate more aggressive therapies.

**Figure 3:**
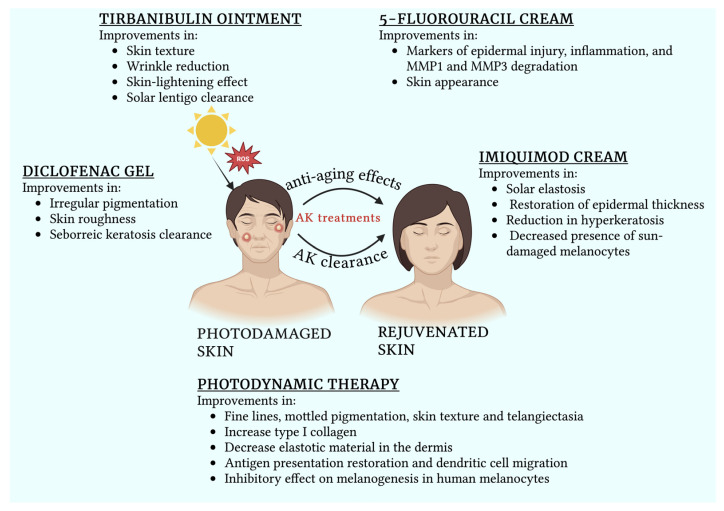
The main anti-aging actions of topical treatments used for actinic keratoses in patients with chronic photodamage. Created with BioRender.com.

Tirbanibulin, the newest topical agent, presents a compelling option for dual-purpose use. Preliminary evidence highlights significant improvements in skin appearance, especially SL clearance, reduction in mottled pigmentation, and enhancement in texture, as confirmed by UV fluorescence imaging [84,85]. Its mild inflammatory profile and shorter treatment duration offer advantages over traditional options. It has been hypothesized that tirbanibulin directly targets keratinocytes, impairing their ability to produce endothelin-1 and stem cell factor, which in turn stimulate microphthalmia-associated transcription factor (MITF) activation. Reduced MITF activation may result in reduced melanin production, which is responsible for the typical hyperpigmentation of SLs [85]. However, its anti-aging mechanisms require further validation with in vivo studies (Figure 3).

The link between AKs and photoaging is well established, as both arise from chronic sun exposure, resulting in DNA damage, oxidative stress, and dysregulation of collagen and elastin in the dermis. Targeting AKs with therapies that address these underlying mechanisms may not only reduce the risk of malignant progression but also reverse or mitigate the signs of aging. Such dual-purpose applications align with the evolving understanding of skin health as a continuum, where proactive interventions can simultaneously address pathological and aesthetic concerns. This approach could redefine AK treatment objectives, integrating lesion clearance with broader skin rejuvenation goals.

Traditionally focused on removing lesions and controlling field cancerization, therapies could also be evaluated for their ability to improve skin quality and texture. This could represent a significant shift in dermatologic therapy from a reactive stance against visible damage to a proactive strategy aimed at improving the overall longevity of the skin by preventing the onset of damage. Looking ahead, the integration of anti-aging outcomes into the evaluation of AK therapies could stimulate innovative research perspectives. Clinical studies designed to measure improvements in skin elasticity, pigmentation, and barrier function alongside AK lesion clearance would provide valuable insights into the broader benefits of these treatments. Moreover, sequentially integrating different treatment regimens, common in clinical practice due to AK’s chronic nature, may simultaneously enhance antineoplastic effects while boosting the anti-aging outcomes, thereby improving patient compliance.

As public awareness of skin health grows, patients are increasingly likely to seek interventions that combine skin damage prevention and treatment with visible cosmetic benefits, fueling the demand for multifunctional therapeutic solutions. The development of comprehensive treatment protocols that emphasize both skin health and rejuvenation may establish a new standard of care. By taking a holistic approach that addresses not only AK clearance but also photoaging improvement, these protocols may provide more meaningful and personalized patient outcomes.

Still, patient compliance requires further clarification. Short-term treatments, such as PDT and tirbanibulin, are more likely to gain patient compliance due to their easy application protocols and inflammatory responses that, although sometimes severe, typically last only a few days. In contrast, treatments with longer durations or those associated with LSRs that are either unpredictable (e.g., IMI cream) or known to be severe and prolonged (e.g., 5-FU cream) may negatively impact their acceptance and applicability in managing photoaging.

## 5. Conclusions

The potential anti-aging benefits of topical therapies for AKs represent a promising and underexplored frontier in dermatology. By addressing the root causes of photodamage while delivering cosmetic improvements, these therapies could revolutionize the management of AKs from a narrowly defined medical intervention to a holistic approach for maintaining youthful, resilient skin.

## Figures and Tables

**Table 1:** Studies about the anti-aging and skin-lightening effects of 5-fluorouracil cream. AK: actinic keratosis; SK: seborrheic keratosis; 5-FU: 5-fluorouracil; 5-FU/SA: 0.5% 5-FU combined with 10% salicylic acid.

Authors	Therapy	Mode of Application	Outcome
Sachs et al. [11]	5% 5-FU cream	Twice daily for 2 weeks	Elevation in markers of epidermal damage, inflammation, and extracellular matrix breakdown were observed in 21AK patients
Guimaraes et al. [12]	5% 5-FU cream vs. peel form	Daily for 4 weeks on 1 forearm and 4 weekly peels on the other	Amelioration in arm skin appearance, assessed in 32 patients
Korgavkar et al. [13]	5% 5-FU cream	Twice daily for 2 to 4 weeks	No difference in photoaging was found between baseline and post-treatment assessments in 932 patients
Sciamarrelli et al. [14]	5% 5-FU cream	Daily, for 28 days.	Improvement in skin appearance in a patient 82-year-old woman
Sciamarrelli et al. [14]	5-FU/SA cream	Daily, for 12 weeks	Improvement in skin appearance in a patient 77-year-old woman
Goodhead et al. [15]	5-FU/SA cream	Daily, for 4 weeks	Complete resolution of SKs in a 65-year-old patient

**Table 2:** Studies about the anti-aging and skin-lightening effects of diclofenac gel. CC: complete clearance; HA: hyaluronic acid; RCM: reflectance confocal microscopy; SK: seborrheic keratosis.

Authors	Therapy	Mode of Application	Outcome
Segurado-Miravalles et al. [18]	Diclofenac 3% in HA	2 months	Improvements in irregular pigmentation and skin roughness, along with improvements in RCM features in 20 patients
Afify et al. [19]	Diclofenac 1%	Twice daily for 8 weeks	Reduction in the area affected by SKs in 30 patients
Aktas et al. [20]	Diclofenac 3% in HA	Twice daily for one month	CC of SK in a 73-year-old man

**Table 3:** Studies about the anti-aging and skin-lightening effects of imiquimod cream. IMI: imiquimod; SL: solar lentigo.

Authors	Therapy	Mode of Application	Outcome
Metcalf et al. [24]	IMI 5% cream	Daily, for 3 months	Amelioration of solar elastosis and restoration of normal epidermal thickness in 26 patients
Altalhab et al. [26]	IMI 5% cream	3 non-consecutive days per week, for 8 consecutive weeks	Anti-aging effect in 72.7% of patients who applied the treatment on the periorbital skin
Smith et al. [25]	IMI 5% cream	3 days per week	Hyperkeratosis reduction and decreased presence of sun-damaged melanocytes in the epidermis in biopsies from 12 patients
Cantisani et al. [27]	IMI 3.75% cream	Daily (2-week treatment cycles with 2-week interval without treatment)	Lightening of the SL in a 75-year-old woman
Di Bartolomeo et al. [28]	IMI 3.75% cream	4 weeks of treatments, followed by a second cycle of 6 weeks after 4 weeks of stop	Lightening of the SL in a 77-year-old woman

**Table 5:** Studies about the anti-aging and skin-lightening effect of tirbanibulin 1% ointment. AK: actinic keratosis; CCR: complete clearance response; PCR: partial clearance response; SL: solar lentigo.

Authors	Therapy	Mode of Application	Outcome
Fidanzi et al. [83]	Tirbanibulin 1% ointment	5 consecutive days	Skin-lightening effect in a 78-year-old male with AKs and SL
Li Pomi et al. [84]	Tirbanibulin 1% ointment	5 consecutive days	Anti-aging effect in 7 patients with a reduction in mottled pigmentation and SLs
Li Pomi et al. [85]	Tirbanibulin 1% ointment	5 consecutive days	CCR in 35% of SLs and a PCR in 50% at the 2-month follow-up, assessed in 42 patients with both AKs and SLs
Kopera et al. [22]	Tirbanibulin 1% ointment	5 consecutive days	Improvement in skin quality measurements from baseline to 57 days, in 26 patients

## Data Availability

Not applicable.

## Abbreviations

The following abbreviations are used in this manuscript:

AK	Actinic keratosis
UV	Ultraviolet
5-FU	5-fluorouracil
PDT	Photodynamic therapy
HA	Hyaluronic acid
LSR	Local skin reactions
CCR	Complete clearance rate
SA	Salicylic acid
SL	Solar lentigo
IMI	Imiquimod
NSAID	Nonsteroidal anti-inflammatory drug
COX-2	Cyclooxygenase-2
RCM	Reflectance confocal microscopy
IFN-α	Interferon-alpha
TNF-α	Tumor necrosis factor-alpha
TLR7	Toll-like receptor 7
MAL	Methyl aminolevulinate
ALA	Aminolevulinic acid
ROS	Reactive oxygen species
MMP	Matrix metalloproteinases
TGF-β1	Transforming growth factor-beta 1
MITF	Microphthalmia-associated transcription factor
NADPH	Nicotinamide adenine dinucleotide phosphate hydrogen
cSCC	Cutaneous squamous cell carcinoma
IPL	Intense pulsed light
PDL	Pulsed-dye laser
GAIS	Global Aesthetic Improvement Scale
OTR	Organ transplant recipient
LPNY	Nd:YAG laser
